# Dual PI3K/mTOR inhibition is required to effectively impair microenvironment survival signals in mantle cell lymphoma

**DOI:** 10.18632/oncotarget.2253

**Published:** 2014-07-25

**Authors:** Laia Rosich, Arnau Montraveta, Sílvia Xargay-Torrent, Mónica López-Guerra, Jocabed Roldán, Marta Aymerich, Itziar Salaverria, Sílvia Beà, Elías Campo, Patricia Pérez-Galán, Gaël Roué, Dolors Colomer

**Affiliations:** ^1^ Experimental Therapeutics in Lymphoid Malignancies Group, Institut d'Investigacions Biomèdiques August Pi i Sunyer (IDIBAPS), Barcelona, Spain; ^2^ Hematopathology Unit, Department of Pathology, Hospital Clínic, Barcelona, Spain; ^3^ IDIBAPS, University of Barcelona, Barcelona, Spain

**Keywords:** NVP-BEZ235, mantle cell lymphoma, invasion, cytokine signaling

## Abstract

Phosphatidylinositol-3-kinase (PI3K)/Akt/mammalian target of rapamycin (mTOR) pathway activation contributes to mantle cell lymphoma (MCL) pathogenesis and drug resistance. Antitumor activity has been observed with mTOR inhibitors. However, they have shown limited clinical efficacy in relation to drug activation of feedback loops. Selective PI3K inhibition or dual PI3K/mTOR catalytic inhibition are different therapeutic approaches developed to achieve effective pathway blockage. Here, we have performed a comparative analysis of the mTOR inhibitor everolimus, the pan-PI3K inhibitor NVP-BKM120 and the dual PI3K/mTOR inhibitor NVP-BEZ235 in primary MCL cells. We found NVP-BEZ235 to be more powerful than everolimus or NVP-BKM120 in PI3K/Akt/mTOR signaling inhibition, indicating that targeting the PI3K/Akt/mTOR pathway at multiple levels is likely to be a more effective strategy for the treatment of MCL than single inhibition of these kinases. Among the three drugs, NVP-BEZ235 induced the highest change in gene expression profile. Functional validation demonstrated that NVP-BEZ235 inhibited angiogenesis, migration and tumor invasiveness in MCL cells. NVP-BEZ235 was the only drug able to block IL4 and IL6/STAT3 signaling which compromise the therapeutic effect of chemotherapy in MCL. Our findings support the use of the dual PI3K/mTOR inhibitor NVP-BEZ235 as a promising approach to interfere with the microenvironment-related processes in MCL.

## INTRODUCTION

Mantle cell lymphoma (MCL) is an aggressive B-lymphoid neoplasm with poor response to conventional chemotherapy and short survival. It is genetically characterized by the chromosomal translocation t(11;14)(q13;q32), which dysregulates cyclin D1 expression. Although cyclin D1 up-regulation is detected in nearly all MCL, it is not sufficient for the development of the disease. Additional chromosome alterations that target genes involved in molecular pathways, such as cell cycle, DNA damage response and cell survival, are frequently found in MCL.[1]

Constitutive activation of phosphatidylinositol 3-kinase (PI3K), Akt and mammalian target of rapamycin (mTOR) is known to confer drug resistance to many types of cancer, including MCL. This pathway integrates signals from extracellular stimuli to regulate fundamental cellular processes, including mRNA translation, cell cycle progression and cell survival.[2] MCL tumors frequently express the inactive phosphorylated form of PTEN, a negative PI3K regulator, thereby contributing to constitutive PI3K signaling.[3] In addition, gene ampliﬁcation of *PIK3CA* (PI3K p110 catalytic subunit alpha) has also been described in MCL.[4]

Selective targeting of PI3K has demonstrated the potential to inhibit this pathway. However, the ﬁrst p110δ isoform-selective PI3K inhibitor, idelalisib (GS-1101), with notable results in indolent non-Hodgkin lymphoma[5] and chronic lymphocytic leukemia,[6] showed modest responses in patients with MCL.[7] Moreover, it has been postulated that the increased expression of PI3K p110α isoform in MCL particularly at relapse might play a role in MCL progression,[8] supporting the use of pan-PI3K inhibitors. Currently, multiple PI3K inhibitors are under clinical investigation.[9] Among them, NVP-BKM120 has shown efficacy both *in vitro*[10-13] and *in vivo*[14] in several malignancies.

Allosteric mTOR inhibitors, which include rapamycin (sirolimus) and its analogues (temsirolimus, everolimus and ridaforolimus), mainly target mTORC1 and also show single agent activity in a range of B-cell malignancies.[15] Temsirolimus was approved by the European Medicines Agency for the treatment of relapsed and refractory MCL, while ridaforolimus and everolimus have been entered in several clinical trials.[16] However, mTORC1-specificity of rapamycin analogues may limit its efficacy due to feedback activation of upstream PI3K signaling, leading to Akt hyperactivation.[17] In this sense, we previously reported that activity of the oral mTOR inhibitor everolimus, is limited by Akt rephosphorylation and that a selective Akt inhibitor overcomes this compensatory reactivation.[18] In this context, dual PI3K/mTOR inhibitors have been synthesized, which, unlike rapamycin and its analogues, are ATP competitive inhibitors and target the catalytic site of both kinases.[9] These dual PI3K/mTOR inhibitors have increased antitumor effects due to their ability to suppress the prosurvival regulatory feedback.[19] NVP-BEZ235 is an orally bioavailable imidazoquinoline derivative that inhibits the kinase activity of PI3K, mTORC1 and mTORC2.[20] It has shown pre-clinical activity against a range of lymphoid malignancies[13;19;21-23] and is currently undergoing phase I evaluation in acute leukemia (NCT01756118). NVP-BEZ235 also exhibited antiproliferative effect in MCL cell lines by downregulating Mcl-1[24] and synergisms with conventional agents.[25;26]

In this study, we compared the therapeutic potential of the novel dual PI3K/mTOR inhibitor NVP-BEZ235 to NVP-BKM120 (pan-class I PI3K inhibitor) and everolimus (mTORC1 inhibitor) in primary MCL samples. Our study supports the view that the concurrent suppression of PI3K and mTORC2, in addition to mTORC1, is likely to be a more effective strategy for the treatment of MCL than single inhibition of these kinases.

## RESULTS

### Superior inhibitory activity of NVP-BEZ235 towards PI3K/Akt/mTOR signaling compared to everolimus and NVP-BKM120 in primary MCL cells

**Figure 1:**
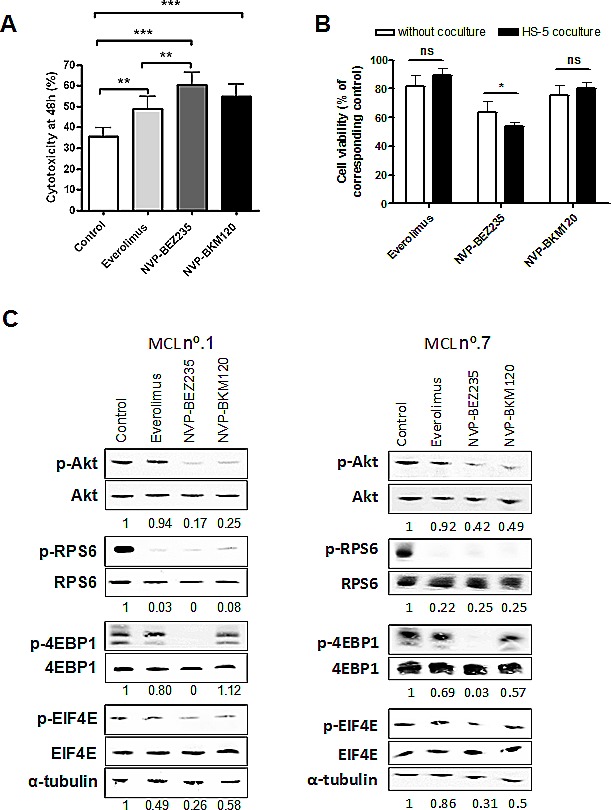
Cytotoxic effect of everolimus, NVP-BEZ235 and NVP-BKM120 and PI3K/Akt/mTOR signaling inhibition in primary MCL cells A, Primary MCL cells were treated with 5 μM everolimus, 1 μM NVP-BEZ235 or 1 μM NVP-BKM120 for 48 hours and cytotoxicity was measured by Annexin V labeling. Mean ± SEM of all the samples analyzed (n=11). B, Primary MCL cells (n=9) were cocultured with or without HS-5 and incubated with the corresponding drugs as above. Cell viability was assessed by Annexin V labeling at 48 hours and calculated relative to the respective untreated control, with or without stroma. Mean ± SEM of the cases analyzed. C, MCL cells were exposed for 8 hours to the corresponding drugs as previously. Analysis of phosphorylated and total levels of Akt, RPS6, 4EBP1 and EIF4E were determined by Western blot. Ratio between phosphorylated and total protein levels was calculated and relative protein quantification in treated versus control extracts was conducted with Image Gauge software (Fujifilm). α-tubulin was probed as a loading control. Two representative cases are shown (MCL nº.1 and nº.7). *, *P* < 0.05; **, *P* < 0.01; ***, *P* < 0.001; ns, not significant.

Cells from 11 primary MCL cases were exposed to everolimus (5 μM), NVP-BEZ235 (1 μM) or NVP-BKM120 (1 μM) for 48 hours and cytotoxicity was measured by Annexin V labeling (Table 1). Drug optimal doses were selected based on previous studies.[10;18;24] These doses showed a low cytotoxic effect in healthy B and T lymphocytes (data not shown). Figure 1A the three compounds induced significant cytotoxicity compared to control (**, *P* < 0.01; ***, *P* < 0.001). Among them, NVP-BEZ235 induced a high cytotoxic effect with a mean response of 40.80 ± 21.30 % which was significantly higher than that observed with everolimus (mean response of 22.74 ± 17.63 %; **, *P* < 0.01). The antitumor effect of the pan-PI3K inhibitor NVP-BKM120 reached 31.93 ± 17.31 %. The sensitivity to these drugs was not related to genomic alterations of PI3K/Akt/mTOR (*PTEN* deletion, *PIK3CA* and *AKT1* amplifications) or *TP53* alterations (Table 1).

**Table 1:** Characteristics of MCL patients

Patient n°	Source	Disease status	Morphologic variant	% of tumor cells^1^	*PTEN*^2^	*PIK3CA*^2^	*AKT1*^2^	*TP53*^2^	% cytotoxicity to everolimus (5μM, 48h)	% cytotoxicity to NVP-BKM120 (1μM, 48h)	% cytotoxicity to NVP-BEZ235 (1μM, 48h)
1	PB	Diagnosis	Classical	95	ND	ND	ND	ND	10.74	36.54	43.26
2	PB	Diagnosis	Classical	92	WT	Gain	WT	WT	23	37.55	70.86
3	PB	Diagnosis	Classical	88	WT	Gain	WT	mut/upd	40.31	33.3	68.96
4	PB	Diagnosis	Classical	92	WT	WT	WT	WT	41.61	55.54	54.21
5	PB	Diagnosis	Classical	94	WT	WT	WT	WT	10.51	6.59	12.17
6	PB	Diagnosis	Classical	80	ND	ND	ND	ND	0	31.28	31.19
7	PB	Diagnosis	Classical	93	ND	ND	ND	ND	10.76	16.31	42.61
8	PB	Diagnosis	Classical	93	WT	Gain	WT	WT	28.44	30.38	31.59
9	PB	Progression	Classical	89	WT	Gain	WT	mut/upd	58.42	61.26	59.21
10	PB	Diagnosis	Classical	96	WT	WT	WT	mut/upd	13.33	35.57	27.77
11	PB	Diagnosis	Classical	97	WT	WT	WT	WT	13.07	6.96	6.94

Abbreviations PB: peripheral blood; ND: not determined; WT: wild type; upd: uniparental disomy; mut: mutation

1 CD19+, CD5+ cells were analyzed by flow cytometry

2 detected by SNP arrays.[43]

We then studied the ability of these drugs to overcome stroma-mediated resistance. As expected, coculture of primary MCL cells with the stromal cell line HS-5 protected MCL cells from spontaneous apoptosis after 48 hours of HS-5 coculture (**, *P* < 0.01; data not shown). Of note, the three compounds were able to induce apoptosis with the same efficiency despite the presence of stromal cells, although only NVP-BEZ235 enhanced MCL cell killing in HS-5 coculture (Figure 1B; *, *P* < 0.05).

We further evaluated the effect of these compounds in the PI3K-mediated signaling. As expected, everolimus blocked the activation of the mTOR downstream target RPS6 but barely modified phospho-Akt levels, consistent with the Akt rephosphorylation after exposure to everolimus.[17;18] We also observed that NVP-BKM120 downregulated the phosphorylation levels of Akt and the mTOR targets, RPS6 and EIF4E, while the dual inhibitor NVP-BEZ235 demonstrated the greater inhibitory activity toward PI3K/Akt/mTOR signaling pathway, with a complete reduction of the phosphorylation levels of Akt, 4EBP1, RPS6 and EIF4E (Figure 1C).

Thus, in MCL primary cells, dual PI3K/mTOR inhibition is the best strategy to efficiently block PI3K-mediated signaling and to induce major apoptosis, even in the presence of stroma.

### NVP-BEZ235 modulates genes related to inflammation, cytokine signaling, angiogenesis and tumor invasiveness

**Figure 2:**
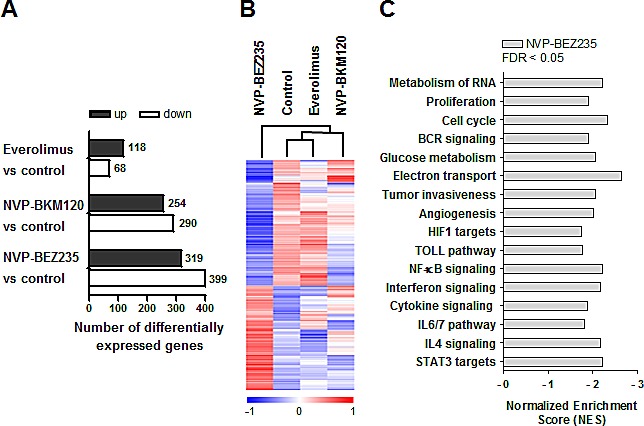
Gene expression profile analysis of primary MCL cells treated with everolimus, NVP-BEZ235 or NVP-BKM120 A, Graph depicting the number of differentially expressed genes from each treatment compared to the control of the two MCL cases, with an absolute fold change > 1.5. B, Heatmap illustrating the 619 exclusively expressed genes modulated by NVP-BEZ235 clustered according to unsupervised hierarchical clustering analysis. Samples with similar patterns of expression of the genes studied cluster together, as indicated by the dendogram. Red indicates increased expression and blue decreased expression relative to the median expression level according to the color scale shown. A representative case is shown (MCL nº.2). C, Gene signatures specifically downregulated by NVP-BEZ235 were obtained with GSEA. Bars represent NES (NES < -1.7). FDR < 0.05 was considered significant.

We next analyzed the impact of these PI3K/Akt/mTOR inhibitors on gene expression profile (GEP) of two representative MCL cases (MCL nº.1 and nº.2, Table 1) treated for 8 hours with the corresponding drugs. We selected the common genes between the two MCL cases that were differentially expressed from each treatment compared to the control, with an absolute fold change above 1.5. Everolimus treatment induced the lowest number of gene modulations (118 genes upregulated and 68 downregulated), whereas after NVP-BKM120 treatment 254 genes were upregulated and 290 genes were downregulated. Interestingly, NVP-BEZ235 modulated the highest number of genes, being 319 genes upregulated and 399 downregulated (Figure 2A). Unsupervised hierarchical clustering in each case showed that control and everolimus-treated samples clustered together, consistent with the low number of genes modified by the drug. NVP-BKM120-treated samples clustered with the previous control-everolimus group. Importantly, NVP-BEZ235-treated samples showed the most different gene expression pattern, as indicated by the independent branch of the dendogram (Supplementary Figure A.1). We then selected the genes specifically modulated by NVP-BEZ235 that were not modified by the other inhibitors. Figure 2B shows the heatmap of these 619 genes (281 genes upregulated and 338 genes downregulated) in a representative primary MCL case.

As NVP-BEZ235 came out to be the compound that induces more dramatic changes in GEP, we next explored the biological networks involved in NVP-BEZ235 treatment in MCL cells. As shown in Figure 2C, using gene set enrichment analysis (GSEA) with a false discovery rate (FDR) < 0.05 and a normalized enrichment score (NES) < -1.7, we identified several gene sets negatively enriched in NVP-BEZ235-treated cells that were related to respiratory electron transport, glucose metabolism, B-cell receptor signaling, cell cycle and proliferation, all of them pathways well-known to be affected by PI3K/Akt/mTOR inhibitors.[2] In addition, we also identified other pathways negatively enriched in NVP-BEZ235-treated cells involved in hypoxia response, angiogenesis, cell invasion, cytokines and inflammation, such as NF-κB pathway, TOLL pathway, IL4 and IL6 signaling, and STAT3 pathway. Complete information of the gene signatures is shown in Supplementary Table A.2.

Altogether, these findings suggest that improved antitumor activity of NVP-BEZ235 in MCL is mostly associated with its capacity to modulate a high number of genes related to angiogenic and invasive processes, inflammation and cytokine signaling.

### NVP-BEZ235 blocks IL4 and IL6 signaling in MCL cells

**Figure 3:**
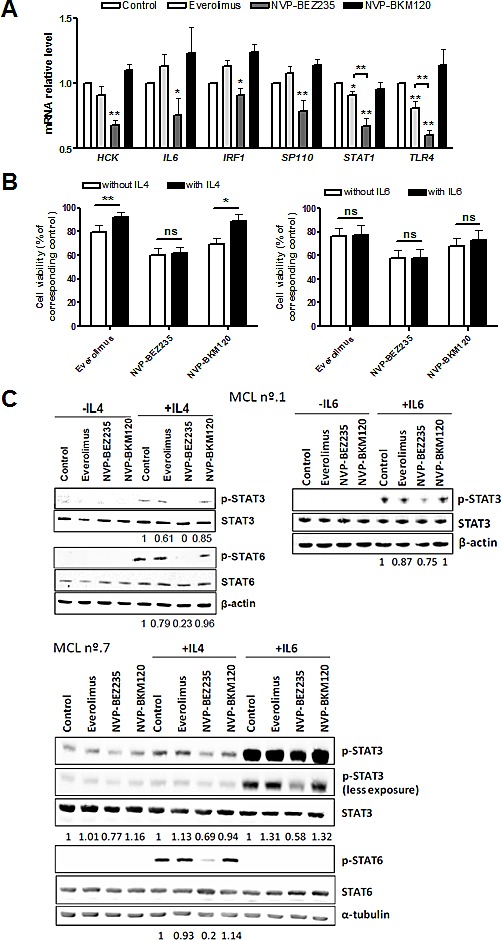
NVP-BEZ235 disrupts IL4 and IL6 signaling A, Primary MCL cells were exposed to 5 μM everolimus, 1 μM NVP-BEZ235 and 1 μM NVP-BKM120 for 8 hours and mRNA levels of the selected genes were examined by qRT-PCR. Relative mRNA expression levels were referred to untreated controls. Bars represent the mean ± SEM of all the samples tested (n=10). B, MCL cells were simultaneously incubated with IL4 (20 ng/mL) or IL6 (40 ng/mL) and the corresponding drugs (5 μM everolimus, 1 μM NVP-BEZ235 or 1 μM NVP-BKM120). Cell viability was determined at 48 hours by Annexin V labeling and represented relative to the respective untreated control, with or without IL4 (n=8) or IL6 (n=7). Mean ± SEM of the cases analyzed. C, Western blot analysis of phosphorylated and total levels of STAT3 and STAT6 was performed after 30 minutes of simultaneous exposure of IL4 or IL6 and the corresponding drugs. β-actin or α-tubulin was probed as a loading control. Two representative cases are shown (MCL nº.1 and nº.7). Ratio between phosphorylated and total protein levels was calculated with Image Gauge software (Fujifilm). *, *P* < 0.05; **, *P* < 0.01; ns, not significant.

In order to validate the relevance of the GSEA gene categories related to inflammation and cytokine signaling, we selected a subset of the top leading edge genes for further analysis in 10 primary MCL cells exposed to the different drugs (Supplementary Table A.1). Several genes related to the IL4, IL6 and STAT3 signatures, *HCK* (**, *P* < 0.01), *IL6* (*, *P* < 0.05)*, IRF1* (*, *P* < 0.05) and *SP110* (**, *P* < 0.01) were validated as being downregulated only by NVP-BEZ235 treatment. *STAT1* was downregulated by both everolimus (*, *P* < 0.05) and NVP-BEZ235 (**, *P* < 0.01), although NVP-BEZ235 effect was more pronounced. Statistical significance was also achieved with *TLR4* (**, *P* < 0.01), from the TOLL pathway signature (Figure 3A).

To further study the effect of NVP-BEZ235 in interleukin signaling, primary MCL cells were exposed to the corresponding drugs in the presence or absence of IL4 or IL6 (Figure 3B). We observed that IL4 protected MCL cells from spontaneous apoptosis (*, *P* < 0.05; data not shown). Interestingly, while the cytotoxic effect of everolimus and NVP-BKM120 was almost completely reverted by IL4 (**, *P* < 0.01 for everolimus; *, *P* < 0.05 for NVP-BKM120), no alteration of NVP-BEZ235 citotoxicity was observed in the presence of IL4. It is known that the activating phosphorylation of STAT proteins can be triggered by cytokines such as IL4 and IL6.[27] As shown in Figure 3C, incubation of primary MCL cells with IL4 resulted in the phosphorylation of downstream STAT6 and, in a minor degree, of STAT3. Consistent with GEP data, NVP-BEZ235 was the only drug able to block STAT6 and STAT3 phosphorylation after IL4 stimulation, indicating that this compound is interfering IL4 signaling in primary MCL cells. In the case of IL6 stimulation, despite no differences in viability were observed after treatment with the corresponding drugs (Figure 3B), only NVP-BEZ235 was able to decrease the phosphorylation levels of STAT3 induced by IL6 (Figure 3C).

These data demonstrate that only the dual inhibitor NVP-BEZ235 is able to hamper IL4 and IL6 signaling in MCL cells compared to everolimus or NVP-BKM120.

### PI3K/Akt/mTOR inhibitors effect on CXCL12-induced MCL cell migration and invasion and tumor angiogenesis

**Figure 4:**
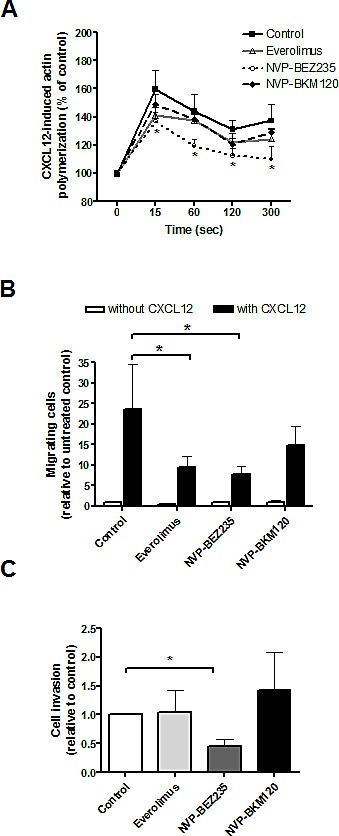
PI3K/Akt/mTOR inhibitors effect on CXCL12-induced MCL cell migration and invasion A, Primary MCL cells were preincubated with 5 μM everolimus, 1 μM NVP-BEZ235 or 1 μM NVP-BKM120 for 1 hour before CXCL12 (200 ng/mL) addition. Polymerized actin content was determined at the indicated time points after CXCL12 addition. Results are displayed relative to the samples (n=7) before chemokine stimulation (100 %). Bars represent the mean ± SEM. B, MCL samples (n=7) were assayed for chemotaxis toward CXCL12 after 1 hour of preincubation with the drugs. Relative number of migrating cells to the untreated control without CXCL12 is represented. Bars correspond to the mean ± SEM. C, Samples (n=7) were assayed for invasion toward CXCL12 through matrigel invasion chambers. Invasion is represented as the ratio between invasive cells and input viable cells, relative to the untreated control. Bars correspond to the mean ± SEM. *, *P* < 0.05.

To further validate GSEA results, we next explored the role of these PI3K/Akt/mTOR inhibitors in invasive and angiogenic processes in MCL. As tumor invasiveness couples with the migratory ability of cells, we first investigated the effect of these drugs on actin polymerization and cell chemotaxis in response to CXCL12. As shown in Figure 4A, CXCL12 induced a notable increase in actin polymerization that was significantly decreased only after NVP-BEZ235 incubation (*, *P* < 0.05). MCL cells were then assayed for chemotaxis toward CXCL12. Figure 4B shows that everolimus and NVP-BEZ235 significantly reduced the number of migrating MCL cells in the presence of the chemokine (59.80 ± 6.87 % of inhibition for everolimus, 66.87 ± 4.78 % of inhibition for NVP-BEZ235; *, *P* < 0.05), whereas NVP-BKM120 had no significant effect. We then examined the effect of these drugs on MCL invasive properties with matrigel-coated invasion chambers that simulate extracellular matrix. In response to CXCL12, untreated MCL cells passed through matrigel, however, NVP-BEZ235 significantly reduced CXCL12-induced invasion (*, *P* < 0.05) whereas NVP-BKM120 and everolimus did not (Figure 4C).

**Figure 5:**
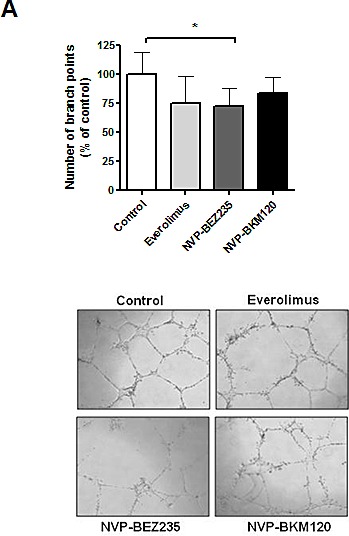
NVP-BEZ235 downregulates angiogenesis in MCL cells A, Supernatants from MCL cells were harvested after 8 hours of incubation with drugs and added to HUVEC cells for 24h. The number of branch points was quantified as the mean of 5 randomly chosen fields from each well, as relative to the untreated control. Bars represent the mean ± SEM of 6 MCL cases. Microscope images (x40 magnification) from a representative case are shown (MCL nº.7). *, *P* < 0.05.

Finally, we analyzed the effect of these drugs on tumor angiogenesis with HUVEC tube formation assay. Supernatants from MCL cells treated with NVP-BEZ235 were significantly less angiogenic than those from untreated MCL cells (*, *P* < 0.05), although MCL cells exposed to everolimus or NVP-BKM120 showed a tendency but not a significant decrease in the angiogenic activity on HUVEC cells (Figure 5A).

All these results show that NVP-BEZ235 exerts a significant reduction of migratory, invasive and angiogenic properties of MCL cells.

## DISCUSSION

MCL is characterized by an unfavorable clinical evolution being one of the most aggressive B-cell lymphomas. Furthermore, current conventional therapies are not able to control the long term evolution of the disease and the tumors present frequent relapses. The constitutive activation of the PI3K/Akt/mTOR signaling pathway, which is involved in the regulation of essential cellular functions, is commonly observed in many tumors, including MCL, and is critical for tumor progression and resistance to antineoplastic drugs.[3;9]

Antitumor activity has been observed with mTOR inhibitors as monotherapy or in combination. However, only a portion of relapsed/refractory MCL patients respond to the rapamycin analogues and the response is generally not durable.[28-32] It has been reported that several signaling feedback loops might attenuate the effectiveness of these compounds,[17] so dual catalytic inhibition of PI3K/mTOR could be a good strategy to address PI3K/Akt/mTOR resistance mechanisms.

Here, we have compared the effect of everolimus, an mTORC1 inhibitor, NVP-BEZ235, a dual inhibitor of PI3K and mTOR (mTORC1 and mTORC2) and NVP-BKM120, a pan-PI3K inhibitor, in primary MCL cells. We observed that NVP-BEZ235 was more powerful than everolimus or NVP-BKM120 in PI3K/Akt/mTOR signaling inhibition and in cytotoxicity induction in the presence of stromal cells. Accordingly, the ability of NVP-BEZ235 to overcome microenvironment signals has been previously reported in MCL cell lines.[25] Our results confirmed for the first time this effect in primary MCL cells. Thus, the use of the PI3K/mTOR dual inhibitor would support the idea that targeting PI3K/Akt/mTOR at multiple levels might be more efficient at inducing apoptosis in the presence of microenvironment signals and to prevent the development of drug resistance. This is in agreement with a previous report of our group, where we showed that dual mTORC1 and Akt targeting with everolimus and an isoselective Akt inhibitor, respectively, exerted synergistic antitumor activity in MCL.[18]

Based on the differences in drug-related cytotoxic effect, GEP approach revealed that NVP-BEZ235 treatment was associated with the highest number of modulated genes compared to NVP-BKM120 and everolimus, being the MTORC1 inhibitor the drug that induced the lowest number of gene changes. Consistently, NVP-BEZ235-treated MCL samples downregulated well-known pathways affected by PI3K/Akt/mTOR inhibitors.[2] In addition, we observed inhibition of pathways involved in angiogenesis, cell invasion and inflammation processes. Importantly, NVP-BEZ235 treatment downregulated IL6 and IL4 cytokine signaling pathways confirming an essential contribution of STAT family to the PI3K/mTOR pathway[33] in MCL.

Interactions between the neoplastic B cells and accessory cells in tissue microenvironments, such as the lymphatic tissues, are critical for disease progression and chemoresistance in various B-cell malignancies.[34] In MCL, STAT3 activation has shown to be either constitutively through a cytokine autocrine loop of IL6 and/or IL10 secretion or in response to B-cell receptor engagement.[35] Moreover, IL6–mediated STAT3 activation has recently been found to compromise the therapeutic effect of chemotherapy in MCL.[36] In this context, we demonstrated that NVP-BEZ235 was able to decrease STAT3 signaling in the presence of IL6, in contrast to everolimus or NVP-BKM120. Besides, NVP-BEZ235 also downregulated the gene expression levels of *HCK*, *IRF1*, *SP110* and *STAT1*, all genes related to the IL6 signaling pathway. As human bone marrow stromal cells have been described to secrete IL6,[37;38] we hypothesize that NVP-BEZ235 increased cytotoxicity in HS-5 coculture could be related to its ability to interfere with cytokine signaling.

Growth-promoting activity of IL4 by means of STAT6 phosphorylation has also been described in B-cell lymphomas.[33;39-41] Interestingly, NVP-BEZ235 effectively overcame the prosurvival effect of IL4 as well as completely inhibited IL4 signaling. In addition, TLR4 levels were also found to be downregulated by NVP-BEZ235 treatment. A previous report described that the activation of TLR4 signaling in MCL cells, one of the predominant TLRs in these cells, induced proliferation and secretion of cytokines like IL6 and VEGF.[42] Recently, a whole-exome sequencing study detected recurrent activating mutations in TLR2 that increased the IL6 production among other chemokines.[43] Moreover, NVP-BEZ235 was found to inhibit multiple paracrine and autocrine survival growth factors in PI3K/Akt/mTOR-addicted lymphomas.[19] Thus, our data support that NVP-BEZ235 treatment may interfere with cytokine signaling in MCL, which is crucial for keeping tumor thriving in its microenvironment.

A key event in the development and progression of cancer is the potential of tumor cells to migrate and invade into surrounding tissues. In this context, we show for the first time that NVP-BEZ235 clearly reduced the migratory and invasive potential of MCL cells, as indicated in GEP data. Recently, ibrutinib[44] and sorafenib[45] have been described to inhibit migration of MCL cells, indicating the potential of the BCR-associated kinase inhibitors in modulating the homing of MCL cells into lymphoid tissues. Besides migratory and invasive processes, angiogenesis appears to be of particular importance in the physiopathology of lymphomas, as disease progression was found to correlate with increased angiogenic activity.[46] In this sense, involvement of PI3K/Akt/mTOR pathway in cell invasion and angiogenesis has been proposed in follicular lymphoma.[47] Here, we provide the first evidence of NVP-BEZ235 ability to interfere with the angiogenic process. This effect could be related to the observed downregulation of STAT3 signaling, as it was demonstrated to participate in angiogenic responses in vivo.[48]

MCL is one of the most difficult types of lymphoma to treat, which is due in part to its frequent relapses and progressive resistance to treatment suggesting that the microenvironment may sustain residual tumor cells resistant to chemotherapy.[1] Our findings suggest the use of NVP-BEZ235 as a therapeutic strategy to interfere with malignant cell/microenvironment interactions, thanks to its involvement in essential processes such as angiogenesis, migration, invasiveness and cytokine signaling. We propose that targeting the PI3K/Akt/mTOR pathway at multiple levels may therefore provide a more effective antitumor activity than the current strategies using mTOR inhibitors in MCL.

## METHODS

### Culture of primary and stromal cells

Primary cells from 11 patients diagnosed of MCL were obtained. The ethical approvals for this project including the informed consent of the patients were granted following the guidelines of the Hospital Clínic Ethics Committee and The Code of Ethics of the World Medical Association (Declaration of Helsinki). The characteristics of these patients are listed in Table 1. The DNA copy number alterations and mutational profile of these samples were previously characterized using Affymetrix 6.0 SNP arrays and whole-exome sequencing.[43] Primary cryopreserved MCL cells were cultured as previously described.[18] When indicated, MCL cells were cocultured with the human bone marrow-derived stromal cell line HS-5 (ATCC) in 96-well plates as described.[10]

### Treatments and flow cytometry analysis of apoptosis

Primary MCL cells were incubated as indicated with 5 μM everolimus, 1 μM NVP-BEZ235 or 1 μM NVP-BKM120 (all drugs kindly provided by Novartis). When indicated, cells were simultaneously incubated with interleukin 4 (IL4; 20 ng/mL) or interleukin 6 (IL6; 40 ng/mL) and the drugs. Cell viability was quantified by flow cytometry analysis after double labeling of cells for phosphatidylserine exposure with Annexin V-fluorescein isothiocyanate (FITC), and for nuclear membrane permeabilization with propidium iodide (PI; eBioscience). Annexin V-FITC and PI negative population was considered as viable cells. Labeled cells were acquired on an Attune cytometer (Life Technologies).

### Western blot analysis

Whole protein extraction and Western blot analysis were done as previously described.[18] Membranes were probed with the following primary antibodies from Cell Signaling Technology (Danvers): phospho-Akt (Ser473), phospho-ribosomal protein S6 (RPS6; Ser235/236), phospho-4EBP1 (Thr37/46), phospho-eIF4E (Ser209), phospho-STAT3 (Tyr705, clone D3A7), phospho-STAT6 (Tyr641), Akt, RPS6, 4EBP1, eIF4E, STAT3 (clone 79D7) and STAT6 (clone D3H4). Horseradish peroxidase-labeled anti-mouse IgG (Sigma) and anti-rabbit IgG (Cell Signaling Technology) were used as secondary antibodies. Ratio between phosphorylated and total protein levels was calculated and relative protein quantification in treated versus control extracts was conducted with Image Gauge software (Fujifilm). Equal protein loading was confirmed by re-probing membranes with anti-β-actin or anti-α-tubulin antibodies (Sigma).

### Gene expression profiling (GEP)

Total RNA was isolated from primary MCL cells, previously exposed to 5 μM everolimus, 1 μM NVP-BEZ235 or 1 μM NVP-BKM120 for 8 hours, using the TRIzol reagent (Life Technologies) according to manufacturer's instructions. RNA integrity was examined with the Agilent 2100 Bioanalyzer (Agilent Technologies) and 150 ng of high quality RNA were used to generate biotin-labeled cRNA. After cRNA fragmentation, samples were hybridized on the HT HG-U219 GeneChip perfect-match-only array plate (Affymetrix) following standardized protocols. Scanning was processed in the Gene Titan instrument (Affymetrix) and analyzed with GeneChip Command Console Software (AGCC) (Affymetrix). Raw data were normalized using the Robust Multichip Analysis (RMA) algorithm implemented in the Expression Console Software v1.1 (Affymetrix). Raw data have been deposited in the Gene Expression Omnibus Database (accession number GSE53309).

Unsupervised hierarchical clustering of the 10000 high-standard deviation genes was performed using Pearson's correlation coefficient and average linkage, as the similarity measure and clustering algorithm respectively, within TM4-MultiExperiment Viewer platform. Genes modulated by a specific treatment were displayed with Cluster (version 2.11) and TreeView (version 1.6) softwares (Eisen Laboratory). The scaled expression value is plotted in red-blue color scale with red indicating high gene expression and blue indicating low gene expression.

### Gene set enrichment analysis (GSEA)

Significant gene signatures differentially regulated by each drug *versus* untreated cells were identified with GSEA version 2.0 (Broad Institute at MIT; http://www.broadinstitute.org/gsea/) using the C2 (curated gene sets) collection from the Molecular Signature Database v2.5. A two-class analysis with 1000 permutations of gene sets and a weighted metric was used. Gene sets with a false discovery rate (FDR) below 0.05 were considered significant.

### mRNA quantification by real-time PCR

Total RNA was extracted using TRIzol method (Life technologies) as above. In order to eliminate any traces of DNA, RNA was incubated with the DNA-free kit (Life Technologies). DNA-free RNA (0.5-1 μg) was retrotranscribed to cDNA using the High Capacity cDNA Reverse Transcription kit (Life Technologies). Then, samples were processed to Specific Target Amplification using the TaqMan PreAmp Master Mix and the TaqMan Gene Expression Assays (Life Technologies) for a multiplexed preamplification of the targets of interest (Supplementary Table A.1). Finally, samples were diluted 1/5 in Tris-EDTA buffer and 48.48 Dynamic Array – Gene Expression IFC (Fluidigm Corporation) was run as recommended by the manufacturer, with the same TaqMan Gene Expression Assays as before. The relative expression of each gene was quantified by the comparative cycle threshold (*C*t) method (*ΔΔC*t) using *GUSB* as endogenous control. mRNA expression levels are given in arbitrary units, taking as a reference the untreated control sample.

### Chemotaxis and actin polymerization assays

CXCL12-induced chemotaxis and actin polymerization assays were performed as previously described.[10] Migration is represented as the relative number of migrating cells, taking as a reference the untreated control without CXCL12. As for actin polymerization assays, results are plotted relative to the mean fluorescence of the sample before the addition of CXCL12.

### Cell invasion assay

Primary MCL cells were serum-starved for 1.5 hours in FBS-free RPMI 1640 (10^7^ cells/mL). Next, cells were diluted (5×10^6^ cells/mL with 0.5 % BSA in RPMI 1640), incubated with the corresponding drugs for 1 hour and afterwards, 100 μL were added to the top chambers of rehydrated BioCoat Matrigel Invasion Chambers (Becton Dickinson). Chambers had been previously transferred to wells containing 600 μL of 0.5 % BSA in RPMI 1640 with 200 ng/mL of CXCL12. Input cell count was obtained from adding 100 μL of cell suspension to wells containing 600 μL of 0.5 % BSA in RPMI 1640. After 24 hours of incubation, 100 μL were collected in triplicate from each lower chamber and input well, viable cells gated in a FSC/SSC plot and counted on an Attune cytometer under constant flow rate. Invasion is represented as the ratio between invasive cells and input viable cells, relative to the untreated control.

### HUVEC tube formation assay

HUVEC cells, kindly provided by Dr MC Cid (IDIBAPS), were cultured in RPMI 1640 supplemented with 20 % defined bovine calf serum (Thermo Fisher Scientific), 0.2 mg/mL endothelial cell growth supplement (Becton Dickinson), 5 units/mL sodium heparin (EMD Millipore), 2 mM glutamine, 100 units/mL penicillin-streptomycin, 50 μg/mL gentamycin (Life Technologies) and 2.5 μg/mL fungizone (Sigma). Supernatant from primary MCL cells (2×10^6^ cells/mL) was collected after 8 hours of incubation with drugs. Twenty-four-well plates were coated with 300 μL of Matrigel (Becton Dickinson) and allowed to polymerize for 30 minutes at 37ºC. Subsequently, 500 μL of the supernatant of interest and 500 μL of HUVEC cells (0.8×10^5^ cells/mL) in its medium were added into each culture well. After 24 hours of incubation, the number of branch points was quantified as the mean of 5 randomly chosen fields from each well. Images were taken at x40 magnification in a DM IL LED microscope coupled to a DFC295 camera with Leica Application Suite v 3.7 software (Leica).

### Statistical analysis

Nonparametric Wilcoxon signed rank test was used to compare the mean of a set of samples to a theoretical value. Comparison between two groups of samples was evaluated by non-parametric Wilcoxon paired t-test. Statistical analysis was conducted with the use of GraphPad Prism 4.0 software (GraphPad Software).

## SUPPLEMENTARY MATERIAL TABLES AND FIGURE


