# Supplementary material for: Dual PI3K/mTOR inhibition is required to effectively impair microenvironment survival signals in mantle cell lymphoma
Source: Oncotarget. 2014 Jul 25;5(16):6788–800. doi: 10.18632/oncotarget.2253 (PMC4196163; doi:10.18632/oncotarget.2253)

Dual PI3K/mTOR inhibition is required to effectively impair microenvironment survival signals in mantle cell lymphoma

Supplementary Material

Table S1. Validation of gene expression profiling by RT-PCR

Gene	Gene sets	mRNA level (relative to control) Everolimus	NVP-BEZ235	NVP-BKM120
HCK	IL6/7 pathway, cytokine signaling	0.91 ± 0.21	**0.68 ± 0.12**	1.10 ± 0.15
IL6	IL4 signaling, IL6/7 pathway, cytokine signaling	1.13 ± 0.31	**0.76 ± 0.40**	1.23 ± 0.65
IRF1	IL6/7 pathway, interferon signaling, cytokine signaling	1.13 ± 0.16	**0.91 ± 0.18**	1.24 ± 0.19
SP110	STAT3 targets	1.08 ± 0.16	**0.79 ± 0.26**	1.14 ± 0.14
STAT1	STAT3 targets, IL6/7 pathway, tumor invasiveness, interferon signaling, cytokine signaling	**0.91 ± 0.1**	**0.67 ± 0.20**	0.96 ± 0.16
TLR4	TOLL pathway	**0.81 ± 0.16**	**0.60 ± 0.13**	1.14 ± 0.38
BCL2L1	IL6/7 pathway	1.09 ± 0.16	0.9 ± 0.2	1.07 ± 0.14
CD69	IL4 signaling	1.15 ± 0.19	1.16 ± 0.38	1.16 ± 0.16
MYD88	TOLL pathway, cytokine signaling	0.99 ± 0.11	1 ± 0.18	1.08 ± 0.12
PPP2CA	IL4 signaling, metabolism of RNA, glucose metabolism	0.97 ± 0.07	1.09 ± 0.21	1.07 ± 0.12
TLR2	TOLL pathway	1 ± 0.35	1.04 ± 0.49	1 ± 0.32

Gene signatures were obtained from GSEA, from the Molecular Signature Database v2.5
Statistically significant results are indicated in bold

Table S2. Gene sets significantly downregulated in NVP-BEZ235-treated samples vs control MCL cells

Gene sets (C2all)	Gene set size	Everolimus NES	Everolimus FDR	NVP-BEZ235 NES	NVP-BEZ235 FDR	NVP-BKM120 NES	NVP-BKM120 FDR
DAUER_STAT3_TARGETS_DN	49	-1.219	0.415	-2.220	2.46E-05	1.430	0.185
LU_IL4_SIGNALING	89	1.133	0.530	-2.170	4.20E-05	1.387	0.209
PID_IL6_7PATHWAY	47	1.131	0.530	-1.810	0.009	1.134	0.421
REACTOME_CYTOKINE_SIGNALING_IN_IMMUNE_SYSTEM	260	-1.003	0.702	-1.870	<1E-05	1.129	0.427
REACTOME_INTERFERON_SIGNALING	151	-1.380	0.258	-2.163	5.09E-05	-1.223	0.349
REACTOME_ACTIVATION_OF_NF_KAPPAB_IN_B_CELLS	61	-0.811	0.916	-2.220	2.43E-05	-1.622	0.064
BIOCARTA_TOLL_PATHWAY	37	-0.972	0.742	-1.760	0.013	-1.007	0.633
SEMENZA_HIF1_TARGETS	34	-1.312	0.314	-1.740	0.016	-1.609	0.068
HU_ANGIOGENESIS_DN	37	0.815	0.933	-2.020	8.05E-04	0.810	0.875
REACTOME_RESPIRATORY_ELECTRON_TRANSPORT	65	-1.752	0.067	-2.630	<1E-05	-1.121	0.464
REACTOME_GLUCOSE_METABOLISM	64	-1.423	0.228	-2.070	3.32E-04	-1.473	0.138
REACTOME_SIGNALING_BY_THE_B_CELL_RECEPTOR_BCR	121	-0.988	0.720	-1.900	<1E-05	-1.578	0.081
REACTOME_REGULATION_OF_MITOTIC_CELL_CYCLE	76	-1.744	0.070	-2.330	<1E-05	-1.649	0.054
BENPORATH_PROLIFERATION	138	-1.216	0.416	-1.910	<1E-05	-1.330	0.241
WANG_TUMOR_INVASIVENESS_UP	364	0.866	0.896	-2.060	3.85E-04	-1.521	0.110
REACTOME_METABOLISM_OF_RNA	249	0.717	0.980	-2.216	2.41E-05	-1.626	0.062

NES, Normalized Enriched Score; FDR, False Discovery Rate
C2all motif gene signatures were obtained from the Molecular Signature Database v2.5

Supplementaty Figure1:

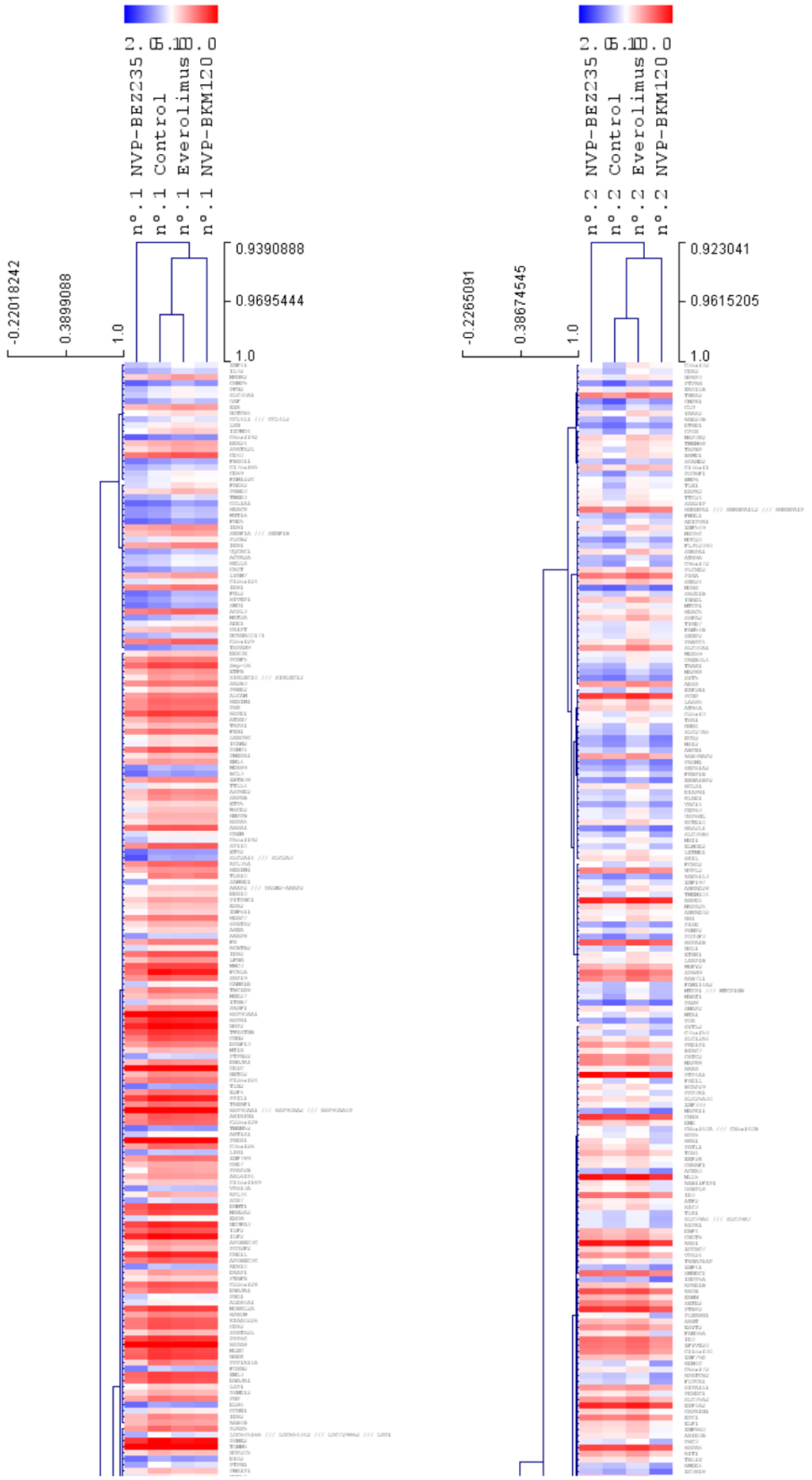

Supplement: Supplementary file 1 [file oncotarget-05-6788-s001.pdf]
